# Green composites based on volcanic red algae *Cyanidiales,* cellulose, and coffee waste biomass modified with magnetic nanoparticles for the removal of methylene blue

**DOI:** 10.1007/s11356-023-26425-3

**Published:** 2023-03-22

**Authors:** Paulina Pietrzyk, Ewa Izabela Borowska, Patrycja Hejduk, Bruno Cury Camargo, Magdalena Warczak, Thu Phuong Nguyen, Agnieszka Pregowska, Marianna Gniadek, Jacek Szczytko, Sławomir Wilczewski, Magdalena Osial

**Affiliations:** 1 Institute of Fundamental Technological Research, Polish Academy of Sciences, Pawińskiego 5B, 02-106 Warsaw, Poland; 2 The College of Inter-Faculty Individual Studies in Mathematics and Natural Sciences (MISMaP), University of Warsaw, Banacha 2C, 02-097 Warsaw, Poland; 3 Faculty of Chemistry, University of Warsaw, Pasteura 1, 02-093 Warsaw, Poland; 4 Institute of Experimental Physics, Faculty of Physics, University of Warsaw, Pasteura 5, 02-093 Warsaw, Poland; 5 Faculty of Chemical Technology and Engineering, Bydgoszcz University of Science and Technology, Seminaryjna 3, 85-326 Bydgoszcz, Poland; 6 Institute for Tropical Technology, Vietnam Academy of Science and Technology, 18 Hoang Quoc Viet, Cau Giay District, Hanoi, 10000 Vietnam; 7 Extremo Technologies, Klecińska 123, 54-413, Wrocław, Poland

**Keywords:** Organic biomass waste, Magnetic nanoparticles, Water treatment, Adsorption, Eco-friendly composite, Green composite

## Abstract

In this paper, green nanocomposites based on biomass and superparamagnetic nanoparticles were synthesized and used as adsorbents to remove methylene blue (MB) from water with magnetic separation. The adsorbents were synthesized through the wet co-precipitation technique, in which iron-oxide nanoparticles coated the cores based on coffee, cellulose, and red volcanic algae waste. The procedure resulted in materials that could be easily separated from aqueous solutions with magnets. The morphology and chemical composition of the nanocomposites were characterized by SEM, FT-IR, and XPS methods. The adsorption studies of MB removal with UV-vis spectrometry showed that the adsorption performance of the prepared materials strongly depended on their morphology and the type of the organic adsorbent. The adsorption studies presented the highest effectiveness in neutral pH with only a slight effect on ionic strength. The MB removal undergoes pseudo-second kinetics for all adsorbents. The maximal adsorption capacity for the coffee@Fe_3_O_4_–2, cellulose@Fe_3_O_4_–1, and algae@Fe_3_O_4_–1 is 38.23 mg g^−1^, 41.61 mg g^−1^, and 48.41 mg g^−1^, respectively. The mechanism of MB adsorption follows the Langmuir model using coffee@Fe_3_O_4_ and cellulose@Fe_3_O_4_, while for algae@Fe_3_O_4_ the process fits to the Redlich-Peterson model. The removal efficiency analysis based on UV-vis adsorption spectra revealed that the adsorption effectiveness of the nanocomposites increased as follows: coffee@Fe_3_O_4_–2 > cellulose@Fe_3_O_4_–1 > algae@Fe_3_O_4_–1, demonstrating an MB removal efficiency of up to 90%.

## Introduction

Water pollution is one of the most challenging environmental issues in the modern world (Rashid et al. 2021). Despite many commonly used water treatment techniques, innovative, cost-effective methods are still deeply needed. Effective wastewater treatment can be performed with many different techniques, including membrane separation (Li 2014; Wu et al. 2012), adsorption (Boi 2016; Nadour et al. 2019; Shao et al. 2008; Phuong et al. 2022; Olusegun et al. 2023a, b), photocatalytic oxidation (Rezaei et al. 2021), electrochemical methods (Alnajrani and Alsager 2020; Spaltro et al. 2020), biopolymer sorption (Zhou et al. 2016; Zhou et al. 2017a, b), photodegradation (Hussain et al. 2020; López Zavala and Espinoza Estrada 2016, Nagamine et al. 2020, Olusegun et al. 2023b), biosorption (Zhou et al. 2019), and biodegradation (Wang et al. 2020). Among them, adsorption-based processes are considered the most effective, also offering cost-effectiveness, simplicity of preparation, and high customizability (Rashed 2013). One of the most commonly used adsorbents is activated carbon. This material exhibits a high active surface area (Zhu et al. 2020; Mariana et al. 2021), enabling a responsive treatment of various types of chemicals in water, both at high and low concentrations. Despite being an outstanding adsorbent, activated carbon is not widely used in sewage treatment—mainly due to the high costs associated with its production and activation (Naganathan et al. 2021). Much effort has been put into finding equally efficient, yet cheaper alternatives. Nowadays, candidates for this role are natural materials called biosorbents, such as algae biomass, coffee grounds, or cellulose waste (Elgarahy et al. 2021). Coffee is the second most consumed beverage in the world (Begum et al. 2021), generating low-cost biomass that can be reused in many fields. It offers a highly porous structure that can effectively act as an adsorbent, while its sorption properties can be further improved by carbonization in strong acids, increasing the material’s porosity (Conway 2021). In particular, activated carbon obtained from coffee residues can be a promising adsorbent for the removal of bisphenol A (Colantoni et al. 2021) and basic Toluidine Blue (Alves et al. 2019) from aqueous solutions. It has found use as a filtering agent for waste-, drinking- and contaminated waters (Gul et al. 2021). Moreover, it demonstrated the capability to trap heavy metals and toxic elements such as cadmium, lead, copper, and arsenic (Lafi et al. 2014; Azouaou et al. 2010; Kyzas 2012). Due to its effectiveness, it can be considered an attractive green and low-cost adsorbent material for the removal of diverse contaminants from water. Cellulose biomass is also widely used in water treatment (Nam et al. 2017; Chavan et al. 2016; Carpenter et al. 2015; Jiang et al. 2021) especially in heavy metals removal including lead, zinc, and cadmium (Li et al. 2021; Idress et al. 2021). Additionally, cellulose-based composites have controllable properties such as adsorption and pollutant degradation rates. Another biomaterial with promising properties in wastewater treatment is algae biomass (Wang et al. 2021; Leong et al. 2021). It can be used as an organic source of biomaterials in filters (Gupta et al. 2021; Katam and Bhattacharyya 2021) and composites (Yadav et al. 2021; Eroglu et al. 2021). Among the many types of algae, microalgae are a low-cost and easily obtainable source of biomass with a large specific surface for the adsorption of different chemical compounds.

Regardless of the high effectiveness in water purification with biomass, it must be effectively removed from decontaminated solutions with filtration or be pre-prepared mechanically as a membrane. When associated with other well-established applications, nanotechnology holds great promise to address this issue in sustainable development. So far, coating biomass with magnetic particles for magnetic separation from water only with magnets can reduce wastewater costs. Besides providing magnetic properties, such an approach significantly improves the adsorption properties of the pristine compounds by enhancing their active surface areas (Arbab et al. 2021; Aragaw et al. 2021). One of the most promising materials for this application, offering a high surface area to volume ratio, non-cytotoxicity, fast kinetics, strong adsorption capacities, and photostability, is iron oxide (Sylvester et al. (2007); Pietrzyk et al. (2022); Olusegun et al. (2021)) making it possible to be applied in different fields, from water remediation to medical diagnostics and even vibration dissipation (Xu et al. 2012; Dave and Chopda 2014; Kaili et al. 2019; Gaweda et al. (2020); Żuk et al. 2021; Nieciecka et al. (2021); Osial et al. 2022). When used as a coat in core-shell composites, it improves the versatility and effectiveness of the adsorbent employed in wastewater treatment (Gutierrez et al. 2017), especially when it comes to the facile magnetic separation employing simple magnets or electromagnets and then reusing it in another treatment cycles (Sharma et al. 2018). Such an approach promotes sustainable development and perfectly matches the circular economy. The feasibility of Fe_3_O_4_-embedded filtering compounds has already been demonstrated in the literature. For example, in ref. (Taylor et al. 2012; Xie et al. 2014), a variety of adsorbent solutions based on iron oxide nanoparticles were reported. Magnetic nanocomposite adsorbents based on magnetite and clay have also been successfully applied for water purification in ref. (Alchouron et al. 2021) and photodegradation of organic pollutants (Liu et al. 2013). In addition, ref. (Na et al. 2021) shows iron oxide-biochar nanocomposites loaded with bacteria as a promising material for bioremediation in wastewater treatment. As the agricultural sector generates a tremendous amount of biomass waste, its effective management is a big issue. Therefore, its application in magnetic sorbents is not only an effective way to recycle agricultural waste but also shows it as a functional material that, thanks to the magnetic coat, simplifies the separation of the adsorbent after the effective treatment.

In this paper, methylene blue (MB) as a cationic dye with widespread use in textiles, was used as a model pollutant. Its disposal in waterways compromises the survival of aquatic wildlife and humans feeding on it since MB contamination can lead to severe health problems (Hakami et al. 2021; Olusegun et al. 2021; Yang et al. 2020; Hameed et al. 2007). Its removal was performed with magnetic separation using biomass coated with iron oxide nanoparticles. Given this, we followed such a field, investigating the effectiveness of novel biowaste-based magnetic adsorbents in removing the model pollutant—methylene blue, from an aqueous solution, where the biomass was coffee, cellulose, and algae waste. The magnetic composite was characterized using scanning electron microscopy (SEM), Fourier transform infrared spectroscopy (FTIR), magnetometry, and X-ray photoelectron spectroscopy (XPS). The adsorption studies were performed using UV-vis spectrometry. Obtained results presented a promising applicability toward water purification.

## Material and methods

### Materials

The raw materials used as adsorbents in the present work were 100% arabica ground coffee obtained from Tchibo, microcrystalline cellulose with a purity ≥ 97% from Merck S.A., and pelleted volcanic algae biomass of the *Cyanidiales* axenic strain (*Cyanidioschyzon merolae*, 10D, obtained from the Microbial Culture Collection at the National Institute of Environmental Studies in Japan), cultivated in low pH 1.5.

Materials used for the growth of magnetic nanoparticles were analytical-grade iron (III) chloride hexahydrate FeCl_3_·6H_2_O Aldrich ACS reagent 97% and iron (II) chloride tetrahydrate FeCl_2_·4H_2_O p.a. ≥ 99%. Both were supplied by Sigma-Aldrich co. Additionally, 25% ammonia solution NH_4_OH was supplied by CHEMPUR, and ethanol 96% was supplied from POCH. Distilled water used during all steps of sample preparation was sourced from Merck’s Milli-Q ultra-pure water filtering system and exhibited a resistivity of 18.2 MΩ·cm at 25 °C. Methylene blue (analytical grade) was purchased from WarChem Sp. z o.o., Poland.

### Composite synthesis

In this work, core-shell magnetic nanoparticles were synthesized through a facile co-precipitation technique. Cores were adsorbents prepared from algae biomass, coffee waste, or cellulose, whereas shells were composed of iron oxide. The ratio between both of these components was chosen to yield nanoparticles with the highest possible efficiency in catching pollutants.

In total, three sets of samples were produced. They are described as follows: the first composite was based on coffee waste. Prior to use, ground coffee was leached in scalding water with a commercial coffee maker operating at a pressure of 14 bars, followed by air-drying on a hotplate at 90 °C for 2 h. The dried powder was stirred mechanically for 15 min in a 1:2 ethanol and water solution, filtered, and dried again at the same conditions.

To coat the coffee residue with iron oxide, 2 g of it was immersed in 10 mL of a solution containing 1.08 g of iron (III) chloride hexahydrate FeCl_3_·6H_2_O and 0.40 g of iron (II) chloride tetrahydrate FeCl_2_·4H_2_O. The mixture was warmed up to 75 °C on a hotplate and stirred with a magnetic bar at a rate of about 1000 rpm to dissolve iron salts. To precipitate the iron oxide onto the organic core, the 25% ammonia solution was added dropwise until pH 10. The suspension was then stirred for 15 min at 75 °C. This process resulted in magnetite-covered ground coffee particles, with a ratio of 2 g of coffee per 570 mg ± 6 mg of Fe_3_O_4_ coat. The mass of iron oxide product on the organic core was estimated based on the weight of the dry powder mass. After the synthesis, the magnetic composite was collected at the bottom of the glass vial with a neodymium magnet and washed with deionized water until a neutral pH was achieved. The solid underwent air-drying on a hot plate at 75 °C for 1 h, followed by 90 °C for 1 h and subsequently 110 °C for 15 min. This drying method ensured slow evaporation of the water content, preventing steam-induced damage to the magnetic coating. The final product, labeled cofee@Fe_3_O_4_–1, was stored in a sealed glass vial prior to characterization.

Another batch of the cofee@Fe_3_O_4_ compound was also prepared with a different coffee: iron oxide ratio. It followed the same procedure outlined above but used 4 g of coffee residue (instead of 2 g). The resulting product, coffee@Fe_3_O_4_–2, had a lesser iron oxide coating onto the organic core than coffee@Fe_3_O_4_–1. The second composite was based on microcrystalline cellulose waste (cellulose@Fe_3_O_4_). The synthesis was performed following to same procedure outlined for cofee@Fe_3_O_4_, but by replacing the washed coffee with as-purchased microcrystalline cellulose. Therefore, to prepare cellulose@Fe_3_O_4_–1 and cellulose@Fe_3_O_4_–2 preparations, 2 g and 4 g of cellulose were used, respectively. As an effect, the ratio between the cellulose and iron oxide was about 4:1 (2 g of cellulose over 570 mg Fe_3_O_4_ coat)—cellulose@Fe_3_O_4_–1 and 8:1 (4 g of cellulose over 570 mg Fe_3_O_4_ coat)—cellulose@Fe_3_O_4_–2. The last composite was based on volcanic algae. Before synthesis, fresh algae were washed in water and centrifuged for several minutes, and then, the biomass was dried on a hot plate at 75 °C for 1 h. The dry biomass was subsequently fine-ground into a powder, where the same procedure of composite synthesis was performed. The ratio between the algae and iron oxide was about 4:1 (2 g of algae over 570 mg Fe_3_O_4_ coat)—algae@Fe_3_O_4_–1 and 8:1 (4 g of algae over 570 mg Fe_3_O_4_ coat)—algae@Fe_3_O_4_–2. The biomass in sorbents was not carbonized—unlike most reports in the literature (Christou et al. 2018). This omission ensured a lower quantity of chemical refuse, thus reducing financial and environmental costs associated with the synthesis process.

### Methods

Before morphology characterization, all composites (before and after coating with Fe_3_O_4_) were dispersed in ethanol at the ratio of 5 mg^.^mL^−1^, drop-casted onto a flat surface, and allowed to dry in a vacuum overnight. Their morphologies were obtained with a scanning electron microscope (SEM)—FE-SEM Merlin (Zeiss, Oberkohen, Germany) equipped with a Gemini II column. The chemical composition of the samples was measured with X-ray photoelectron spectroscopy (XPS) using a PHI 5000 VersaProbe (ULVAC-PHI) spectrometer with monochromatic Al K*α* radiation (hν = 1486.6 eV). XPS spectra were collected with a hemispherical analyzer at a pass energy of 23.5 eV and 0.1 eV resolution. Magnetic properties were investigated with a quantum design magnetic property measurement system (MPMS XL-7), on magnetic fields below 7 T and in the temperature range 2 K < T < 300 K. UV-vis spectrometry was employed to evaluate chemical concentrations in aqueous solutions. Absorption spectra were recorded using a Perkin Elmer Lambda 35 spectrophotometer.

## Results and discussion

### Morphological analysis

**Fig. 1:**
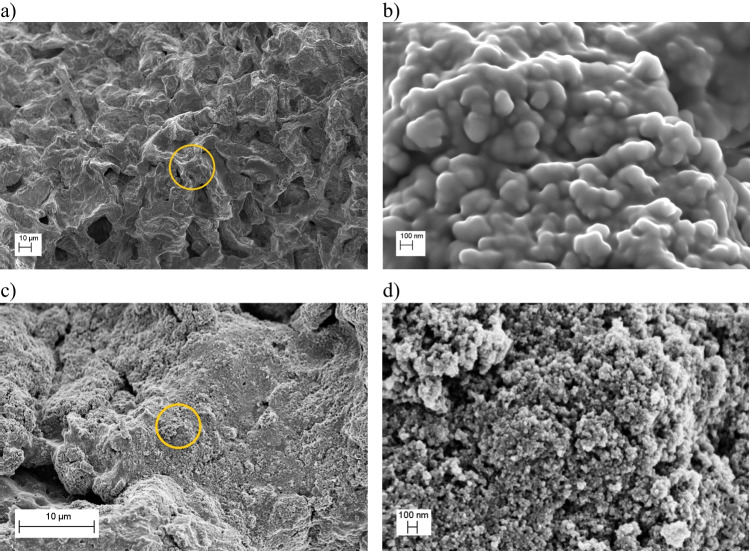
Morphology of coffee@Fe_3_O_4_ composite synthesized from solutions containing **a**,** b** 2 g and **c**,** d** 4 g starting quantities of coffee. The orange ring in the left column corresponds to the region magnified in the right column

The morphology of the composites was investigated with scanning electron microscopy. Samples prepared using 2 g of waste and 570 mg of iron oxide (X@Fe_3_O_4_–1, X = coffee, cellulose, or algae) presented morphologies composed of large surfaces with smooth contours. Meanwhile, samples prepared by using 4 g of biomass over 570 mg of iron oxide (X@Fe_3_O_4_–2) revealed non-homogeneous morphology and differences in the samples with an increase in the waste mass. As can be seen in Fig. 1a for coffee@Fe_3_O_4_–1, the magnetic oxide uniformly covered the whole surface of the coffee grains. This was denoted by rough agglutinated spherical structures, as shown in Fig. 1b. The composite coffee@Fe_3_O_4_–2 exhibited a less homogeneous iron oxide coating. Instead, they revealed a rich structure composed of nanometric magnetic nanostructures adhered to the adsorbent (see Fig. 1c, d).

**Fig. 2:**
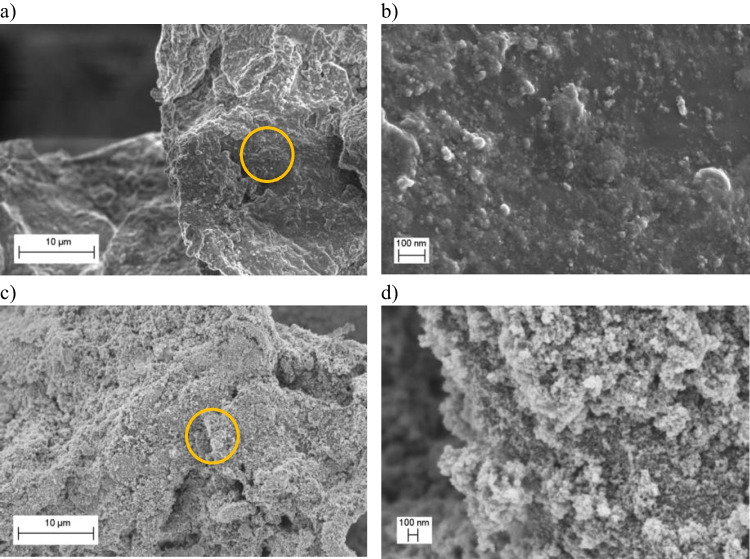
Morphology of cellulose@Fe_3_O_4_ composite synthesized from solutions containing **a**,** b** 2 g and **c**,** d** 4 g starting quantities of cellulose. The orange ring in the left column corresponds to the region magnified in the right column

SEM micrographs obtained for cellulose@Fe_3_O_4_ samples are presented in Fig. 2. Results revealed significantly different morphologies in comparison with bare cores. In the composite cellulose@Fe_3_O_4_–1, the iron oxide formed a smooth film over the organic adsorbent, with few irregularities (see Fig. 2a, b). Particles synthesized with larger cellulose; iron oxide ratios exhibited less homogeneous coatings, see Fig. 2c, d for cellulose@Fe_3_O_4_–2. As can be seen, iron oxide formed irregular clusters of nanoparticles across the adsorbent’s surface.

**Fig. 3:**
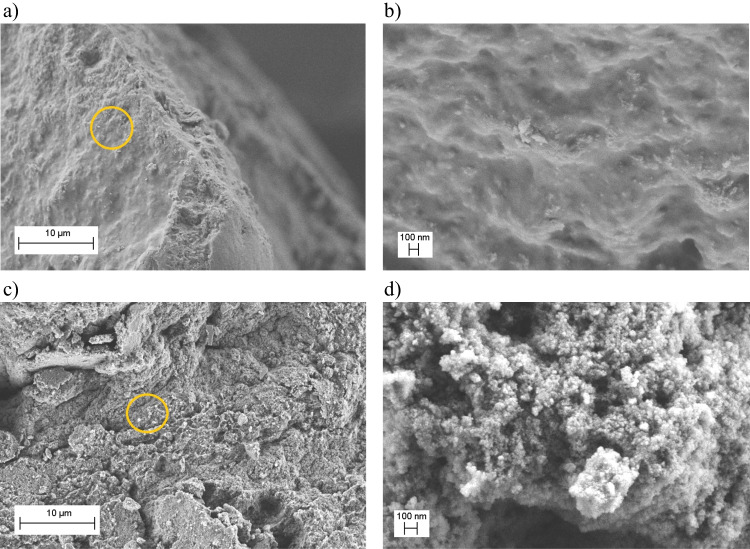
Morphology of algae@Fe_3_O_4_ composite synthesized from solutions containing **a**,** b** 2 g and **c**,** d** 4 g starting quantities of algae. The orange ring in the left column corresponds to the region magnified in the right column

In the composite algae@Fe_3_O_4_–1 (Fig. 3a, b), iron oxide formed a smooth layer on the algae surface. This can be caused by the morphology of the microalgae, as this raw adsorbent had a much smaller size in comparison to the remaining ones. For algae@Fe_3_O_4_–2 (Fig. 3c, d), on the other hand, the overall features of the iron oxide coating were similar to those found on the other composites. Namely, clusters of nanoparticles with typical sizes below 100 nm formed, increasing the composite’s specific surface and improving its potential as a pollutant adsorbent.

Based on the morphology studies, it is seen that the adsorbent offering the largest surface is coffee@Fe_3_O_4_-2, cellulose@Fe_3_O_4_-1, and algae@Fe_3_O_4_-1, so these samples were used for the adsorption studies.

### FT-IR and XPS chemical composition studies

**Fig. 4:**
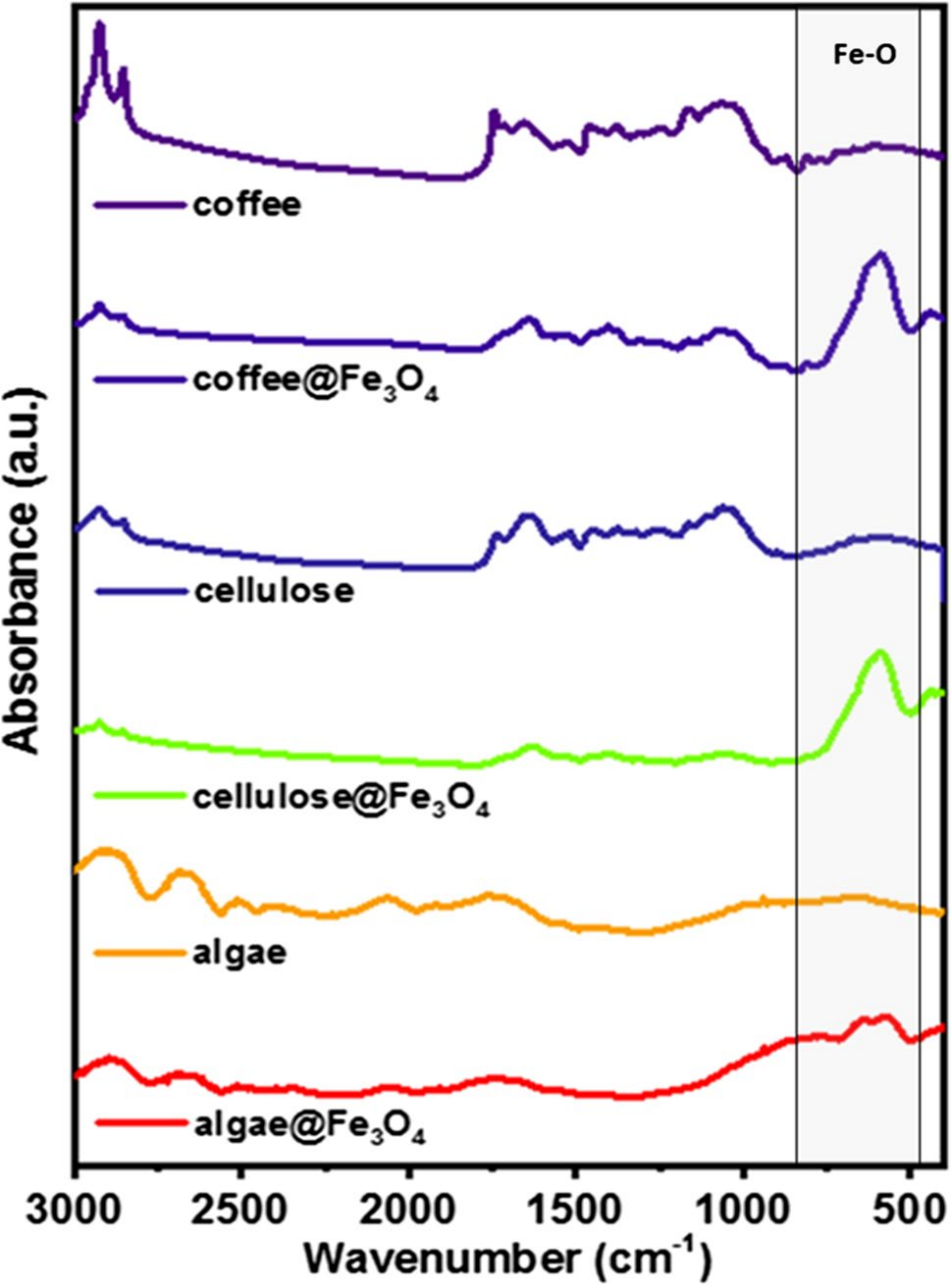
FT-IR spectra for adsorbent cores and their composites. Curves correspond to (from top to bottom): coffee, coffee@Fe_3_O_4_–2 cellulose, cellulose@Fe_3_O_4_–1, algae, and algea@Fe_3_O_4_–1 composite

After the morphological studies, composites were characterized through Fourier transformed infrared spectroscopy (FT-IR). This allowed the identification of the most important functional groups included in our specimens confirming the chemical composition of the samples. Results are shown in Fig. 4. They revealed that all iron oxide-coated adsorbents presented similar spectra, composed by a superposition of characteristic peaks of Fe–O lattices and an attenuated background due to the sample core. In it, the peak at about 2925 cm^−1^ corresponds to the C-H stretching of aliphatic or even aromatic acids, whereas the one at 1745 cm^−1^ can be assigned to the carboxylic groups. The peak at 1713 cm^−1^ stands for either aliphatic acids or ketones, and the one at 1654 cm^−1^ can relate to the C-O stretching and cis C-C bonding (Christou et al. 2018). The presence of the carboxylic group (COO-) is confirmed by a peak at 1527 cm^−1^. Wide peaks appearing at the range 2500–3000 cm^−1^ are attributed to the O-H valence vibrations of carboxylic acids with hydrogen bridges and can be seen in the spectra of algae. The peaks below 650 cm^−1^ can be ascribed to the Fe‐O vibrations in the SPIONs’ lattice (Lesiak et al. 2019).

**Fig. 5:**
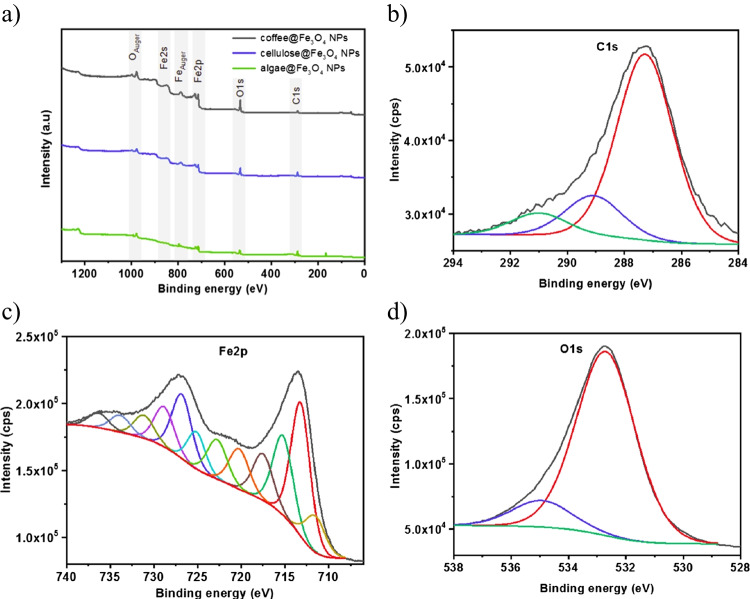
**a** X-ray photoelectron spectroscopy measurements of the composites (from top to bottom): coffee@Fe_3_O_4_–2, cellulose @Fe_3_O_4_–1, and algae@Fe_3_O_4_–1. XPS spectra of bands correspond to **b** C1s, **c** Fe2p, and **d** O1s. The particular bands are described in the text

As X-ray Photoelectron Spectroscopy (XPS) can provide information on the elemental composition of the adsorbent surface and its chemical valence state, it was used complementary to the FT-IR analysis. The spectra presented in Fig. 5a show the chemical binding states of the iron oxide nanoparticles. The survey spectra for all three composites were similar and did not depend on the iron oxide: adsorbent ratio during sample synthesis. Peaks corresponding to C 1 s are ascribed to the organic core, while Fe 2p corresponds to the iron oxide. Due to the latter being located in the NP shell, the Fe peak intensity is much higher than that of carbon. Figure 5b shows a representative XPS measurement of the C 1 s band of the organic absorbents, achieved by probing the coffee-based material. The obtained spectrum is broad and can be deconvoluted into three peaks, associated with different chemical bonds of carbon. Among them, particular peaks marked in different colors in the spectrum can be distinguished. The peak centered at 287.2 eV can be assigned to the C–C or C-H bonds. Meanwhile, the ones centered at 289.3 eV and 291.0 eV can be ascribed to C-N and O = C–OH, respectively (Chickos 2018). Figure 5c shows an XPS spectrum of Fe 2p, revealing two peaks at 713.0 eV and 727.0 eV. These binding energies can be deconvoluted into peaks that correspond to Fe-O bonds characteristic of Fe_3_O_4_ which is made of FeO^.^Fe_2_O_3_.The spectrum for Fe is composed of Fe^2+^ and Fe^3+^, where the valence state increases with the binding energy (Biesinger et al. 2011; Wilson and Langell 2014). The satellite peaks for both valent states are also presented within the spectrum. Results for iron compounds were identical to those presented, which confirmed the formation of iron oxide onto the organic core and will not be shown here. Lastly, the spectrum presented in Fig. 5d is associated with the O 1 s orbital, where the main peak corresponds to the oxygen, that is, the iron oxide lattice. The lower peak can be ascribed to the presence of oxygen in the organic core, relating to the bonds that are also visible at C 1 s (Lesiak et al. 2019), see Fig. 5a.

### Investigation of magnetic properties

**Fig. 6:**
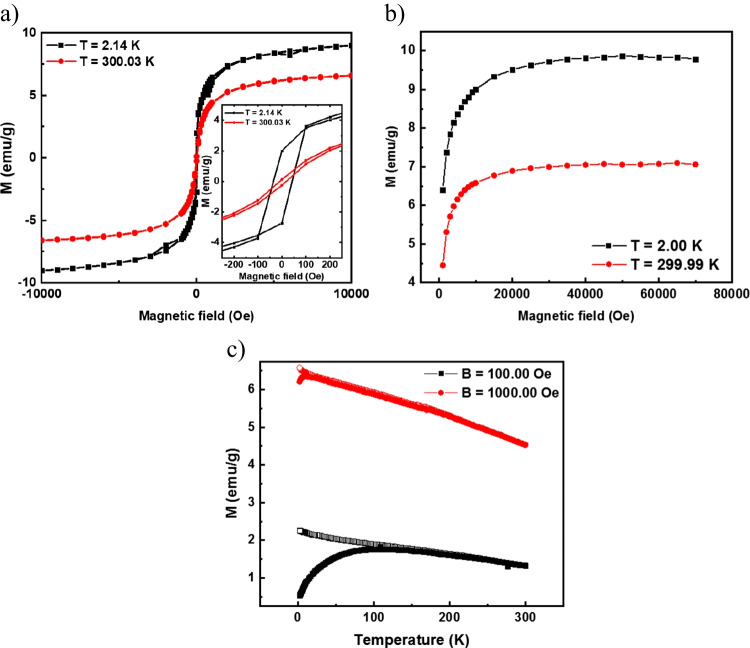
**a** M(H) hysteresis loops obtained for coffee@Fe_3_O_4_-2 at T = 2 K (black squares) and T = 300 K (red circles). No discernible coercive field is observable; **b** M(H) measurements up to the highest available field. Saturation occurs above B ~ 2000 Oe and **c** M(T) measurements on coffee@Fe_3_O_4_. Data was obtained in the ZFC (filled symbols) and FCC (empty symbols) regimes

Magnetic properties of biomass-based nanocomposite were determined by magnetic moment measurements in the temperature interval 2 K < T < 300 K and magnetic fields H up to 70 kOe. Figure 6a, b shows that the M(H) curves revealed a saturation magnetization at T = 300 K around 7 emu^.^g^−1^, which increased with the reduction of temperature up to 9.8 emu^.^g^−1^ at T = 2 K. The inset presented in Fig. 6a reveals very low coercivity of the prepared sample. As the magnetic separation of the proposed materials is an important feature in the presented studies, a deep investigation of the material was performed, including the temperature dependence of magnetization following zero-field-cooled (ZFC) and field-cooled on cooling (FCC) protocols. In the former, the sample was cooled at zero magnetic fields and measured upon warming, whereas in the latter, the measurements occur while cooling the material. Results revealed a paramagnetic, glassy-like behavior that did not obey the Curie-Weiss law up to 300 K (Coey 2009). A hysteresis between the ZFC and FCC curves was present in measurements performed at H = 100 Oe but was almost completely suppressed for fields as small as 1 kOe. The presence of a maximum on M(T, H = 100 Oe) curves at T ~ 100 K could indicate that the compound is composed of a disordered array of magnetic centers with a collective blocking temperature of around 100 K (Fig. 6c).

Despite presenting many inferior properties to pristine magnetite (Kemp et al. 2016), the magnetism of the composites studied here suffices for their application toward the magnetic separation of pollutants.

### UV-vis studies

The effectiveness of the composites in adsorbing methylene blue (MB) pollutants from an aqueous solution was investigated through UV-vis spectrometry. Measurements were performed as a function of the composite amount in solution and the time of exposure to pollutants. The largest effectiveness was achieved for the composites coffee@Fe_3_O_4_–2, cellulose@Fe_3_O_4_–1, and algae@Fe_3_O_4_–1. UV-vis spectra were recorded in the wavelength range from 700 to 200 nm in a quartz cuvette.

**Fig. 7:**
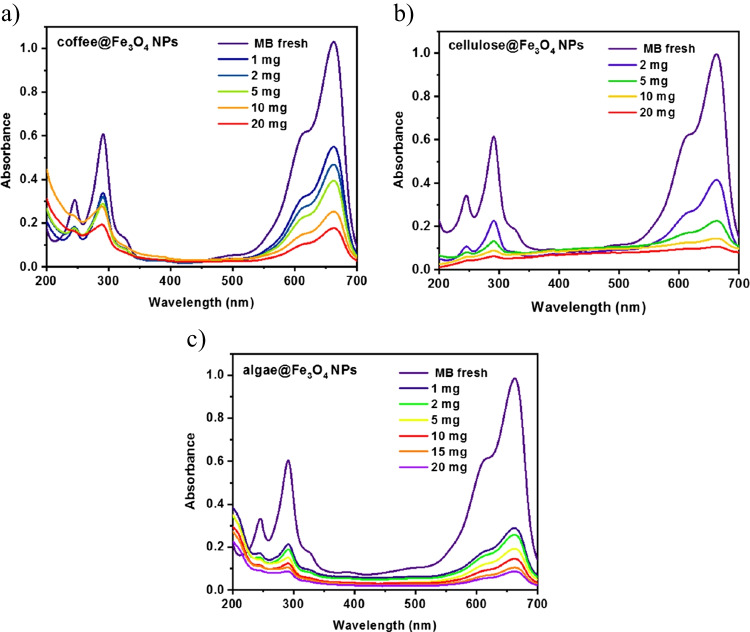
UV-vis spectra of adsorption MB, where the used mass is in the range 1–20 mg for composites: **a** coffee@Fe_3_O_4_, **b** cellulose@Fe_3_O_4_, and **c** algae@Fe_3_O_4_

**Fig. 8:**
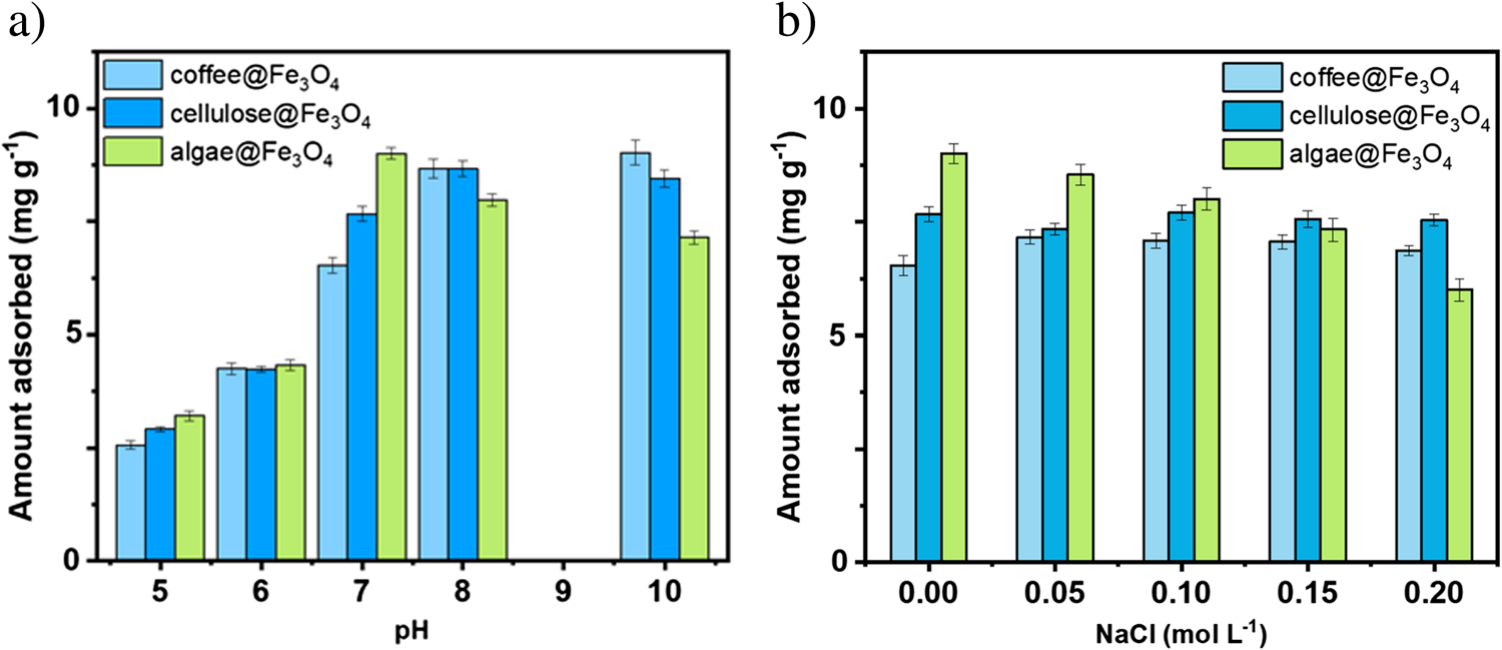
MB amount adsorbed by composited in the function of **a** pH and **b** ionic strength

First, the studies were performed in function of adsorbent mass, where 1, 2, 5, 10, and 20 mg were added to 20 mL of 10 ppm (40 μmol) MB solution with a contact time of about 10 min. Figure 6a–c shows dependence on the used mass of the composites in the adsorption, where the highest % efficiency was obtained for 20 mg of adsorbent. Therefore, such a mass was chosen for the determination of the optimal conditions in the subsequent studies. The time applied for analysis was 10 min. The spectra were normalized to an absorbance equal to 1 (Fig. 7).pH effect and ionic strength are some of the most important variables, which influence pollution adsorption because the wastewater can have different pH and salinity. Figure 8a shows the amount of adsorbent with different pH range from 5 to 10, where the amount of adsorbent was calculated by using Eq. 1:

1 $$Q=\frac{{(C}_{0}-{C}_{t})\times V}{m}$$

where *C*_*0*_ is the initial concentration of dye (mg^.^g^−1^), *C*_*t*_ is the concentration of dye in time (mg^.^g^−1^), *V* is the volume (L), *m* is the mass of adsorbent (g), and *Q* is the amount of adsorbent (mg^.^g^−1^).

The removal of MB by composites increased with the rise of pH value. The highest value was obtained in pH 7 for algae but pH 8 for other composites, which were applied for kinetic studies because of the smallest differences between materials. The subsequent measurements were performed with different concentrations of NaCl in pH 7. Figure 8b shows the increase of ionic strength prompted by to decrease in the amount of adsorbent, especially for algae but composite based on cellulose and coffee displayed similar effectiveness in the MB adsorption process.

**Fig. 9:**
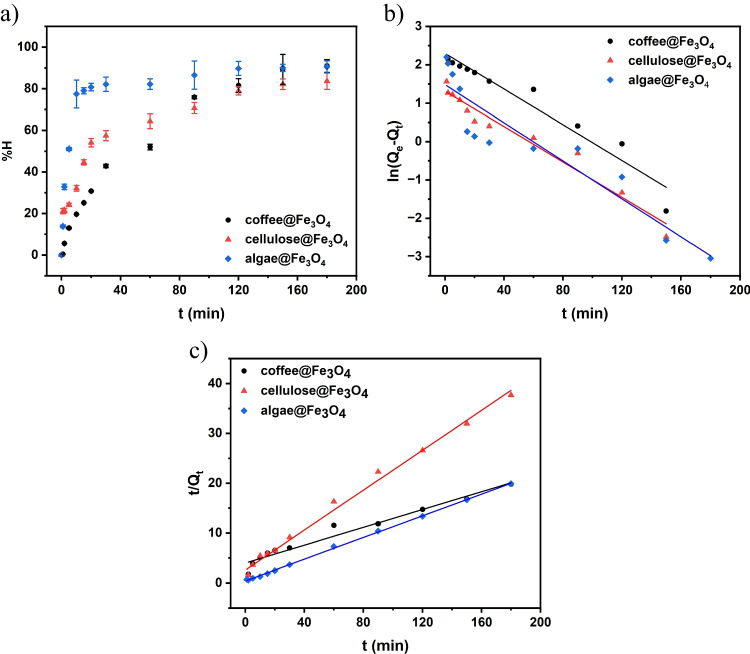
**a** Efficiency of adsorption as a function of time, **b** adsorption data modeled using kinetic Lagergren’s pseudo-first-order, and **c** adsorption data modeled using kinetic McKay and Ho’s pseudo-second-order law

Figure 9a shows the adsorption of MB removal efficiency as a function of time. The process was guided by 180 min; within this time, composites made of algae and coffee, %H efficiency overstepped the 90%, were observed, but for cellulose composite, the removal of dye was reduced compared to other composites. The concentration of dye after the adsorption process was calculated from Eq. 2:

2 $$\%H=({C}_{0}-{C}_{t}/{C}_{0})\times 100\%,$$

where *C*_*t*_ is the MB concentration at time *t* (mg^.^L^−1^), and *C*_*0*_ is the initial MB concentration (mg^.^L^−1^).

Next, the measurements were performed in function of contact time. Based on the obtained results, it can be seen that the 20 min is sufficient to reach over 80% adsorption efficiency %H for algae@Fe_3_O_4_-1, while the elongation of the contact time leads to even higher effectiveness of about 90%, see Fig. 9a. For the coffee waste-based adsorbent, similar effect is achieved after 180 min exposition. (Banat et al. 2003) shows a similar effect, where MB rapidly attaches the adsorbent attaches surface, which is followed by a much slower process associated with the rough surface of iron oxide NPs (Banat et al. 2003). The cellulose-based adsorbent also requires longer contact time than the coffee-based one, while H% values are lower than those of the other composites. Based on the measured removal of the pollutant in contact time, order of the adsorption process has been determined, so in the following studies, MB was treated for 180 min with the proposed magnetic adsorbents.

Adsorption kinetics was characterized by tracking the intensity of spectrophotometric absorption peak 662 nm as a function of time. The kinetics of the pseudo-first adsorption process of MB molecule to a surface can be described through the following Eq. 3:

3 $$\mathrm{ln}\left({Q}_{e}-{Q}_{t}\right)=ln{Q}_{e}-{k}_{1}t$$

where *Q*_*t*_ stands for the amount of adsorbed material per unit mass of adsorbent at a time *t*, whereas *Q*_*e*_ corresponds to its value at equilibrium (Franca et al. 2009). The parameter *k*_*1,2*_ (e.g. *k*_*2*_) is known as the rate constant for pseudo kinetic order adsorption.

For pseudo-second-order kinetic, it follows Eq. 4:

4 $$t/{Q}_{t}=1/{k}_{2} {{Q}_{e}}^{2}+t/{Q}_{e}$$

To determine the kinetics, the values of correlation coefficients *R*^2^ were compared. Figure 9b shows the fitting to the pseudo-first-model kinetics, while Fig. 9c demonstrates the fitting to the pseudo-second-order. Parameters from the fitting linear equation and kinetic parameters have been presented in Table 1. Based on the obtained results, it is seen that the adsorption process follows pseudo-second kinetic order for all composites. The *Q*_e_ values for cellulose and algae composites were similar due to the equilibrium conditions; however, for coffee@Fe_3_O_4_, these results differ, suggesting that such material requires a longer time to achieve equilibrium during MB adsorption.

**Table 1:** Parameters from linear fitting kinetic orders

Composite	k_1_^.^10^−3^ (min^−1^)	*Q*_exp_ (mg g^−1^)	*R*^2^ I kinetic order	*k*_2_^.^10^−3^ (g mg^–1^ min^–1^)	*Q*_exp_ (mg g^−1^)	*R*^2^ II kinetic order	*Q*_cal._ (mg g^−1^)
Coffee@Fe_3_O_4_	23.26	9.9577	0.9323	1.9972	9.0300	0.9591	11.1907
Cellulose@Fe_3_O_4_	23.03	3.7113	0.9538	15.477	4.7777	0.9914	4.9945
Algae@Fe_3_O_4_	24.77	4.3915	0.8601	27.838	9.0492	0.9993	9.2072

As the effectiveness of MR removal differs for particular sorbents, the adsorption isotherms were subsequently measured, providing information about the adsorbent equilibrium performance. The isotherms were estimated for the different concentrations of model pollutants. The initial concentration was changed within the range of 20–180 mg L^−1^ for MB in pH 7 for algae but for coffee and cellulose in pH 8. Experimental data were fitted into the non-linear equations of Langmuir (Eq. 5), Freundlich (Eq. 6), and Redlich-Peterson (Eq. 7) adsorption isotherms (Fig. 9).

5 $${Q}_{e}= \frac{{Q}_{\mathrm{max}}{K}_{L}{C}_{e}}{1+{K}_{L}{C}_{e}}$$

6 $${Q}_{e}= {K}_{F}{{C}_{e}}^{1/n}$$

7 $${Q}_{e}=\frac{{K}_{RP}{C}_{e}}{1+{a}_{RP}{C}_{e}^{g}}$$

where ${C}_{e}$ stands for the equilibrium concentration (mg L^−1^), ${Q}_{e}$ is the equilibrium adsorbed (mg g^−1^), ${Q}_{\mathrm{max}}$ is the maximum adsorption capacity (mg g^−1^), ${K}_{L}$ is Langmuir equilibrium constant (L mg^−1^), ${K}_{F}$ is the Freundlich constant (mg g^−1^ (mg L^−1^)^−1/nF^), ${n}_{F}$ is the dimensionless exponent of Freundlich, where K_RP_ (L g^−1^) and *a*_*RP*_ (mg L^−1^)^−g^, g is an R-P dimensionless parameter (Wu et al. 2010).

**Fig. 10:**
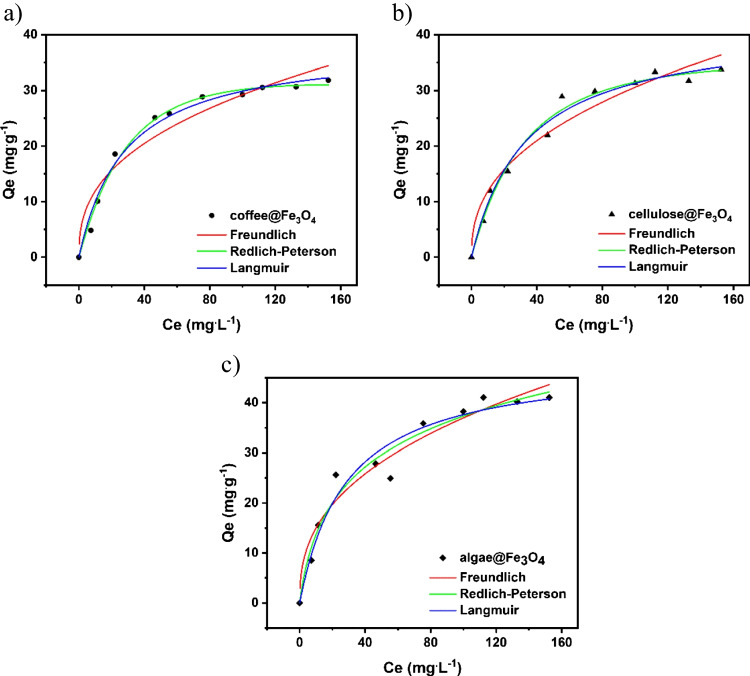
Adsorption isotherms for the MB removal with a) coffee@Fe_3_O_4_-2, b) cellulose@Fe_3_O_4_-1, and c) algae@Fe_3_O_4_-1

Figure 10 shows the non-linear fitting of the adsorption isotherms for the MB removal for all adsorbents. The correlation coefficient *R*^2^ values for all adsorbents are the highest for Langmuir and Redlich-Peterson models. However, the value of $g$ factor is higher than 1 for coffee and cellulose-based adsorbents which excludes the Redlich-Peterson model. Therefore, it can be assumed that the cellulose@Fe_3_O_4_ and coffee@Fe_3_O_4_ follow the Langmuir model. At the same time, the g factor for the algae@Fe_3_O_4_ is lower than 1, suggesting the best fitting to the Redlich-Peterson model, see Table 2, presenting the obtained parameters of isotherms. The adsorption capacity of MB on the surface of used composites: algae@Fe_3_O_4_ > cellulose@Fe_3_O_4_ > coffee@Fe_3_O_4_.

**Table 2:** Isotherms parameters for the MB removal

Isotherms parameters	Composites		
	Coffee@Fe_3_O_4_	Cellulose@Fe_3_O_4_	Algae@Fe_3_O_4_
Langmuir			
* Q*_max_	38.2301	41.6712	48.1113
* K*_*L*_	0.0357	0.0301	0.0347
* R*^2^	0.9855	0.9822	0.9518
Freundlich			
* K*_*F*_	4.8621	4.6044	6.0743
* N*	2.5678	2.4329	2.5502
* R*^2^	0.8866	0.9190	0.9128
Redlich-Peterson			
* K*_*RP*_	1.0557	1.0722	2.7074
* a*_*RP*_	0.0089	0.0136	0.1520
* G*	1.2241	1.1239	0.8072
* R*^2^	0.9907	0.9814	0.9531

Table 3 has shown compared values of obtained adsorption capacity with other nanoparticles and composites. Obtained values of *Q*_max_ are comparable with some materials demonstrated in the literature.

**Table 3:** Compared obtain adsorption capacity with others materials

The material used for MB adsorption	Q_max_ [mg g^−1^]	References
Activated carbon prepared from *Delonix regia* pods	24.00	(Ho et al. 2009)
Activated carbon prepared from wood apple shell	36.90	(Malarvizhi and Sulochana 2008)
Spent coffee grounds	18.73	(Franca et al. 2009)
Composite material made of algae	74.00	(Vilar et al. 2007)
Kaolin	52.76	(Mouni et al. 2018)
Raw ball clay	34.652	(Auta and Hameed 2012)
Graphene-like carbon material	20.0	(Lingamdinne et al. 2018a)
Graphitic carbon-like material	38.75	(Lingamdinne et al. 2018b)
Magnetic fly ash	9.27	(Ahmed et al. 2022)
Expanded graphite/Fe_3_O_4_	78.06	(Wu et al. 2021)
Magnetic chitosan nanocomposite	20.408	(Rahmi and Irfan 2019)
Bio-silica	52.60	(Olusegun et al. 2021)
8%-S-Monolith	10.80	(Kaya-Özkiper et al. 2022)
Coffee@Fe_3_O_4_	38.23	This work
Cellulose@Fe_3_O_4_	41.61	This work
Algae@Fe_3_O_4_	48.41	This work

## Conclusions

In summary, composites based on organic cores and iron oxide shells can be synthesized by the co-precipitation method as an effective way for biomass waste reuse. The obtained magnetic sorbents can be easily used for the removal of dyes from aqueous solutions, which are considered emerging aqueous contaminants and have a potentially harmful impact on living organisms. As a proof of concept, in this paper, green composites based on biomass waste were proposed for the biosorption and removal of methylene blue from water—a potential pollutant widely applied in the chemical and textile industries. The iron-oxide coating ensured a subsequent simple removal of the exhausted filtering media from the solution by magnetic means. All composites presented in this study (coffee@Fe_3_O_4_, cellulose@ Fe_3_O_4_, and algae@ Fe_3_O_4_) were successfully used as supportive materials for the removal of contaminants from water with magnetic sorbent. However, their effectiveness was dictated by the ratio between the core adsorbent and its iron oxide coating. It was found that the degree of activity of our specimens was strictly related to the type of organic adsorbent and the morphology of the composite. For coffee@Fe_3_O_4_, the highest effectiveness was achieved by an iron oxide-to-core ratio of 8:1 (coffee@Fe_3_O_4_–2), whereas for the remaining composites, the ratio was 4:1 (cellulose@ Fe_3_O_4_–1 and algae@ Fe_3_O_4_–1). The adsorption studies showed that the highest effectiveness of the MB removal was near neutral pH, while the presence of salt in the solution slightly affected the process. Kinetic studies showed a pseudo-second-order adsorption process for all compounds. Despite a higher core-shell ratio needed for effective MB removal from solution, the coffee@Fe_3_O_4_–1 and algae@Fe_3_O_4_–1 removed compounds at a higher yield, thus showing greater promise as composites for wastewater treatment. This may be due to the large iron oxide-specific surfaces achieved on these adsorbent cores. The Langmuir isotherm was found suitable to explain the MB adsorption process using coffee@Fe_3_O_4_ and cellulose@Fe_3_O_4_, while for algae@Fe_3_O_4_ the best model is Redlich-Peterson. The maximal adsorption capacity for the sorbents is as follows: 38.23 mg g^−1^ for coffee@Fe_3_O_4_, 41.61 mg g^−1^ for cellulose@Fe_3_O_4_, and 48.41 mg g^−1^ for algae@Fe_3_O_4_, respectively. The broad access to waste organic biomass, the use of magnetic nanocomposites based on natural biosorbents, and the employment of iron oxide to remove organic pollutants from aquatic systems seem to be an economically and technologically practical solution to solving a major global issue. We expect our results to encourage further research activity on the subject, including the synthesis of magnetic core/shell composites with other, locally available, green core materials for wastewater treatment.

## Data Availability

The datasets used and/or analyzed during the current study are available from the corresponding author on a reasonable request.
